# Transglycosylated Starch Improves Insulin Response and Alters Lipid and Amino Acid Metabolome in a Growing Pig Model

**DOI:** 10.3390/nu9030291

**Published:** 2017-03-16

**Authors:** Monica A. Newman, Qendrim Zebeli, Eva Eberspächer, Dietmar Grüll, Timea Molnar, Barbara U. Metzler-Zebeli

**Affiliations:** 1 Institute of Animal Nutrition and Functional Plant Compounds, Department for Farm Animals and Veterinary Public Health, University of Veterinary Medicine Vienna, 1210 Vienna, Austria; Monica.Newman@vetmeduni.ac.at (M.A.N.); Qendrim.Zebeli@vetmeduni.ac.at (Q.Z.); 2 Anaesthesiology and Perioperative Intensive Care, Department for Companion Animals and Horses, University of Veterinary Medicine Vienna, 1210 Vienna, Austria; eva.eberspaecher@vetmeduni.ac.at; 3 Agrana Research & Innovation Center GmbH, 3430 Tulln, Austria; Dietmar.GRUELL@agrana.com (D.G.); Timea.MOLNAR@agrana.com (T.M.); 4 University Clinic for Swine, Department for Farm Animals and Veterinary Public Health, University of Veterinary Medicine Vienna, 1210 Vienna, Austria

**Keywords:** pig model, modified starch, metabolome, metabolite profiles, meal tolerance test, insulin

## Abstract

Due to the functional properties and physiological effects often associated with chemically modified starches, significant interest lies in their development for incorporation in processed foods. This study investigated the effect of transglycosylated cornstarch (TGS) on blood glucose, insulin, and serum metabolome in the pre- and postprandial phase in growing pigs. Eight jugular vein-catheterized barrows were fed two diets containing 72% purified starch (waxy cornstarch (CON) or TGS). A meal tolerance test (MTT) was performed with serial blood sampling for glucose, insulin, lipids, and metabolome profiling. TGS-fed pigs had reduced postprandial insulin (*p* < 0.05) and glucose (*p* < 0.10) peaks compared to CON-fed pigs. The MTT showed increased (*p* < 0.05) serum urea with TGS-fed pigs compared to CON, indicative of increased protein catabolism. Metabolome profiling showed reduced (*p* < 0.05) amino acids such as alanine and glutamine with TGS, suggesting increased gluconeogenesis compared to CON, probably due to a reduction in available glucose. Of all metabolites affected by dietary treatment, alkyl-acyl-phosphatidylcholines and sphingomyelins were generally increased (*p* < 0.05) preprandially, whereas diacyl-phosphatidylcholines and lysophosphatidylcholines were decreased (*p* < 0.05) postprandially in TGS-fed pigs compared to CON. In conclusion, TGS led to changes in postprandial insulin and glucose metabolism, which may have caused the alterations in serum amino acid and phospholipid metabolome profiles.

## 1. Introduction

Rapidly digestible starches, which are consumed in relatively high proportions in the form of processed foods, may contribute to the increasing prevalence of chronic health conditions such as type 2 diabetes, obesity, and insulin resistance [1]. Although these chronic health conditions can be managed via dietary and lifestyle changes, their overall prevalence continues to rise [2,3]. Non-compliance with recommended dietary and lifestyle changes may be a contributing factor, as convenience and taste are the greatest determinants of food selection [4]. Chemically modified starches (CMS) are already commonly used in many processed foods to improve the texture and rheological properties of food products [5,6]. Therefore, considerable interest lies in the development of less digestible CMS to partially replace rapidly digestible starches in processed foods, as they may be able to promote certain health benefits without modifying food selection. However, the health-promoting properties of candidate CMS have not been thoroughly investigated when compared with other starch types [1].

Starches can be chemically modified in different ways, including transglycosylation, esterification, or crosslinking [6]. It is not clear if different CMS elicit similar physiological responses; therefore, the effects from one CMS cannot be assumed for another and any potential health benefits need to be investigated individually. For instance, the capability of various types of CMS such as enzymatically modified starch, hydroxypropyl-distarch phosphate, and cross-linked resistant starch to reduce blood glucose and insulin diverged [7,8,9]. Furthermore, little is known about the effects of CMS on protein metabolism. Because blood insulin is known to affect protein metabolism [10], it is likely that protein metabolism would be affected by the consumption of certain CMS. Although one available study showed that an enzymatically modified starch altered the amino acid metabolome in growing pigs [7], to our knowledge no other data are available regarding the effects of different CMS on protein metabolism. On the other hand, lipid metabolism has shown to be more consistently affected by dietary inclusion of CMS in pigs, humans, and rodents [7,8,11]. Particularly, dietary inclusion of modified starches has been shown to significantly increase fat utilization postprandially in healthy humans [8], and to attenuate a postprandial raise in serum lipids while altering the metabolism of sphingolipids and phospholipids in pigs in the pre- and postprandial state [7]. In mice, significantly lower body weight and visceral fat were observed together with a significantly increased capacity for hepatic fatty acid oxidation [11]. These effects on lipid metabolism could have potential benefits on weight management, especially when combined with a reduction in energy digestibility.

Transglycosylated starch (TGS), a CMS, contains glycosidic bonds that are unable to be broken via host endogenous enzymes in the gastrointestinal tract, theoretically allowing for a more exact and defined reduction in digestibility. However, its effects have not been thoroughly evaluated in vivo. Since targeted blood metabolomics enables the simultaneous identification and quantification of a relatively large number of metabolites, it allows for a much deeper understanding of dietary interventions on systemic metabolism and underlying biological processes. Therefore, in this study a meal tolerance test (MTT) was conducted and a targeted metabolomics approach was used to investigate the effects of TGS on serum protein and lipid metabolome in the pre- and postprandial phase as well as on blood glucose, insulin, and lipids over time compared to rapidly digestible waxy cornstarch in a short-term study using jugular vein-catheterized growing pigs. Additionally, apparent total tract digestibility was investigated to predict the effects of TGS inclusion on starch digestion. We hypothesized that TGS would reduce total tract digestibility of energy and postprandial insulin and glucose responses, thereby altering serum lipid and amino acid profiles. Pigs were used as a model for humans in this study because they are regarded as a reliable model to study digestive physiology and metabolic responses [12,13].

## 2. Materials and Methods

### 2.1. Ethical Statement

All procedures involving animal handling were approved on 24 April 2013 by the institutional ethics committee of the University of Veterinary Medicine Vienna (Vienna, Austria) and the national authority according to to paragraph 8 of Law for Animal Experiments, Tierversuchsgesetz (TVG) (GZ 68.205/0051-II/3b/2013).

### 2.2. Animals, Housing, and Surgery

Eight purebred castrated male growing pigs (Large White; initial BW = 26.1 ± 1.17 kg) were used in this study. One week prior to surgeries, pigs were moved into 1.0 × 1.2 m individual metabolism pens for an environmental adaptation period, where they remained for the duration of the study. Pens, which were cleaned daily, were comprised of Plexiglas walls and completely slatted flooring. Additionally, pens were each equipped with a single-space feeder and a nipple drinker for ad libitum access to demineralized water throughout the study. The room temperature was maintained at 21 ± 1 °C, and was checked twice daily to ensure optimal temperature for the pigs.

Pigs were surgically fitted with a 1-m-long polyethylene catheter (TYGON S-54-HL Medical Tubing; inner diameter 1.016 mm; outer diameter 1.778 mm; Saint-Gobin, Akron, OH, USA) in the jugular vein as described by Metzler-Zebeli et al. [7]. Cardiovascular and respiratory parameters as well as body temperature were monitored and kept constant during surgery. Once fully recovered from anesthesia, pigs were returned to their pens. Catheters were flushed aseptically on a daily basis with 5 mL of 25 IU heparinized normal saline to maintain their patency. Pigs received an analgesic (Metacam; 0.4 mg/kg BW; meloxicam; Boehringer Ingelheim, Ingelheim, Germany) and antibiotic (Cobactan; 2 mg/kg BW; cefquinon sulfate; Intervet GesmbH, Vienna, Austria) one day prior to surgery, on the day of surgery, and for 3 days post-surgery. During the 5-day recovery period following surgery, pigs consumed a commercial grower diet (metabolizable energy (ME) = 3.23 Mcal/kg; crude protein (CP) = 16.3%, as-fed basis). Pigs were not fed on the day of surgery, and feed amounts were gradually increased after surgery until they reached pre-surgery levels about 2 days post-surgery.

Upon completion of the study, pigs were anesthetized via intramuscular injection (Narketan, 10 mL/kg body weight; ketamine HCl; Vétoquinol AG, Ittigen, Austria; and Stresnil, 3 mL/kg body weight; azaperone; Biokema SA, Crissier, Switzerland) and euthanized by intracardiac injection with T61 (10 mL/kg; embutramide; MSD Animal Health, Vienna, Austria).

### 2.3. Diets

Two semi-purified experimental diets based on purified cornstarch, casein, lignocellulose (FibreCell M1; agromed Austria GmbH, Kremsmünster, Austria), rapeseed oil, vitamins, and minerals were fed (Table 1). Diets were formulated to meet or exceed current nutrient requirements for growing pigs [14]. Ingredient composition of the diets was identical, with the exception of the starch component. The control diet (CON) utilized a rapidly digestible waxy cornstarch (Agrana Research and Innovation Center GmbH (ARIC), Tulln, Austria), whereas in the test diet (TGS) 50% of the native waxy cornstarch was replaced by transglycosylated waxy cornstarch (ARIC). The TGS product was prepared via an acid-catalyzed transglycosylation of native waxy cornstarch, which rearranges the glycosidic bonds that are present. Native waxy cornstarch has two types of glycosidic bonds, α(1,4) and α(1,6). The acid-catalyzed transglycosylation of the waxy cornstarch results in the TGS product having eight types of glycosydic bonds: α(1,2), α(1,3), α(1,4), α(1,6), β(1,2), β(1,3), β(1,4), and β(1,6). The analyzed nutrient composition of the diets is presented in Table 1.

Experimental diets were fed at approximately 3 times the estimated energy required for maintenance based on the pigs’ average body weight at the start of each replicate period [14]. This was done to equalize nutrient intake among all pigs. Feed allowance was divided into 2 equal meals fed at 08:00 and 16:00 daily and mixed with water at a ratio of about 2:1.

### 2.4. Experimental Design and Sample Collection

Following the recovery period, pigs were randomly allotted to 1 of the 2 dietary treatments (CON or TGS) according to a complete crossover design with two 8-day replicate periods. Four pigs were allotted per diet in each of the 2 replicate periods, which provided a total of 8 observations per dietary treatment. Each replicate period consisted of 7 days acclimation to diets followed by an 8-hour meal tolerance test (MTT) on Day 8. The dietary acclimation period was similar to previous studies regarding the short-term effects of resistant starch on blood metabolites in growing pigs [7,15]. On Day 7, fresh fecal samples were collected in the morning and evening via grab sampling and were stored at −20 °C until later analysis.

Since it is known that meal size the evening before can affect results of a MTT [16], it was ensured that pigs consumed their entire meal the afternoon prior as well as on the morning of the MTT, which they did freely. Furthermore, all pigs consumed the entire meal portion within 30 min of it being offered. After consuming their meal at 16:00 on the day prior to the MTT, pigs were fasted overnight for 15 h to obtain fasting blood samples used as a baseline measurement. Serial blood samples were taken at −30 (fasting), 30, 45, 60, 90, 120, 150, 180, 210, 240, 300, 360, 420, and 480 min postprandially. Twenty milliliters of blood was drawn for the baseline sample, and 12 mL was drawn for all other blood samples. Blood samples were collected in serum tubes (S-Monovette 9.0 mL Z; Sarstedt AG & Co., Nümbrecht, Germany) for metabolome profiling, triglycerides, cholesterol, NEFA, urea, insulin, and SCFA analyses, as well as in fluoride-EDTA tubes (S-Monovette 2.7 mL FE; Sarstedt AG & Co., Nümbrecht, Germany) for glucose and lactate analyses. Fluids were replenished after each blood sampling with the respective volume of sterile physiological saline (Fresenius Kabi Austria GmbH, Graz, Austria) and catheters were subsequently flushed with 2 mL of 5 IU/mL heparinized saline to prevent clots from forming. All sample collection tubes were immediately placed on ice before centrifugation at 1811× *g* for 20 min (Eppendorf Centrifuge 5810 R, Eppendorf, Hamburg, Germany). Plasma and serum were then divided into aliquots and frozen at −80 °C for metabolomics and insulin analyses, and at −20 °C for all other analyses.

### 2.5. Analytical Methods

#### 2.5.1. Proximate Analyses

After conclusion of the experiment, fecal samples were homogenized, lyophilized (Gamma 2–20, Martin Christ Gefriertrocknungsanlagen GmbH, Osterode am Harz, Germany), and ground through a 0.5 mm screen (GRINDOMIX GM200, Retsch GmbH, Haan, Germany) prior to chemical analyses. Diet samples were also homogenized and ground prior to analyses. Feed and fecal samples were analyzed in duplicate for dry matter, protein, and ash, and feed samples were additionally analyzed for calcium and phosphorus using standard methods of the Association of German Agricultural Analytic and Research Institutes [17]. Gross energy of feed and feces was measured using an isoperibolic bomb calorimeter (C200, IKA^®^-Werke GmbH & Co. KG, Staufen, Germany), with benzoic acid as the standard used to calibrate the instrument. Titanium dioxide was measured in feed and feces according to the method described by Khol-Parisini et al. [18]; absorption was measured at 405 nm using a spectrophotometer (Hitachi U-3000, Metrohm INULA GmbH, Vienna, Austria). To measure total starch content of the TGS diet, the soluble TGS was leached out of the samples and treated with perchloric acid. The resulting glucose was measured using HPLC (Ultimate 3000, Thermo Fisher Scientific, Waltham, MA, USA) equipped with an Aminex HPX-87H separation column and a Refratomax 520 detector. Total starch content of the control diet was measured via the UV method (BOEHRINGER MANNHEIM/R-BIOPHARM, Enzymatic BioAnalysis) using a spectrophotometer (DR 2800, Hach Lange GmbH, Vienna, Austria).

#### 2.5.2. Metabolome Profiling

Metabolites were measured using a targeted metabolomics approach in serum samples collected in the fasting state (−30 min), as well as at 30 and 420 min postprandially. Measurements were conducted using the Absolute*IDQ* p180 kit (BIOCRATES Life Sciences AG, Innsbruck, Austria), which was performed by the Target*IDQ* Service of BIOCRATES Life Science AG as described by Metzler-Zebeli et al. [7]. In brief, this kit is based on electrospray ionization liquid chromatography–mass spectrometry, and allows the simultaneous identification and quantification of 188 endogenous metabolites from 10 μL of blood serum, including amino acids (*n* = 21), biogenic amines (*n* = 21), acylcarnitines (*n* = 40), diacyl (*n* = 38) and acyl-alkyl (*n* = 38) phosphotidylcholines, lysophosphotidylcholines (*n* = 14), sphingomyelins (*n* = 15), and sum of hexoses (*n* = 1). The method of the Absolute*IDQ*™ p180 kit has been proven to be in conformance with the FDA-Guideline “Guidance for Industry—Bioanalytical Method Validation (May 2001)” [19], which implies proof of reproducibility within a given error range. Measurements were performed as described in the manufacturer’s manual UM-P180. The p180 kit is fully automated and run with internal standards. Identification of targeted metabolites was performed using mass spectrometry (4000 QTRAP system; Applied Biosystems/MDS Sciex, Foster City, CA, USA). Chromatograms were analyzed using the BIOCRATES software (BIOCRATES Life Science AG, Innsbruck, Austria) and internal standards were used as reference to calculate metabolite concentrations.

#### 2.5.3. Biochemical Variables

Plasma glucose and lactate, as well as serum triglycerides, cholesterol, urea, and NEFA were measured via standard enzymatic colorimetric analysis using an autoanalyzer for clinical chemistry (Cobas 6000/c501; Roche Diagnostics GmbH, Vienna, Austria). Serum insulin was measured using a commercially available ELISA kit according to the manufacturer’s instructions (Mercodia Porcine Insulin ELISA, Mercodia AB, Uppsala, Sweden). Serum SCFA (acetate, propionate, butyrate, isobutyrate, valerate, isovalerate, and caproate) were measured using a procedure modified from Brighenti et al. [20]. Serum samples were thawed on ice, a 400 μL aliquot of serum was mixed with 50 μL of internal standard (4-methyl-valeric acid; Sigma-Aldrich, Vienna, Austria; [21,22]) and 32 μL of 25% phosphoric acid, homogenized with a vortex, incubated in a waterbath at 60 °C for 30 min, and centrifuged at 8000× *g* for 30 min at room temperature (Avanti^TM^ 30 Centrifuge, Beckman Coulter, Indianapolis, IN, USA). Supernatants were then stored at −80 °C in sealed GC vials. On the day of analysis, supernatants were thawed on ice and analyzed for SCFA using gas-liquid chromatography (GC 8000 series, Fisons Instruments, Ipswich, UK). The GC was equipped with an automatic sampler (AS 800, Fisons Instruments), a flame-ionization detector, and a 30 m × 0.530 mm × 0.53 μm capillary column (Trace TR Wax; Thermo Fisher Scientific, Waltham, MA, USA). Helium was used as the carrier gas with a flow rate of 6 mL per minute. Samples (1 μL) were injected in splitless mode, and standards (1 μL) were injected after every three samples. All samples and standards were injected in duplicate. Concentration and volume of the standards can be found in Table S1. Injector and detector temperatures were 170 °C and 190 °C respectively. The column temperature was initially 65 °C, then was increased to 170 °C at a rate of 15 °C/min, to 190 °C at a rate of 35 °C/min, and finally to 200 °C at a rate of 40 °C/min and held at this temperature for 2 min. Graphs were generated using Stratos Software (Stratos Version 4.5.0.0; Polymer Laboratories, Church Stretton, UK). Identification of the SCFA was based on the retention times of the standard compounds, and final concentrations of each SCFA was calculated with a response factor using the following equations:

Response factor = (concentration of SCFA in sample (μmol) × peak area of internal standard)/(peak area of SCFA in sample × concentration of internal standard (μmol)).
(1)

SCFA in GC vial (μmol) = (peak area of SCFA in sample × response factor × concentration of internal standard (μmol))/peak area of internal standard.
(2)

SCFA in sample (μmol/mL) = SCFA in GC vial (μmol)/sample volume in GC vial (mL)
(3)

### 2.6. Calculations and Statistical Analyses

Apparent total tract digestibility coefficients were calculated for dry matter, gross energy, crude protein, and crude ash according to Oresanya et al. [23].

Area under the curve (AUC) values were computed using the trapezoidal rule in SAS (version 9.3; SAS Inst. Inc., Cary, NC, USA) from 0–480 min postprandially. This method was used to approximate the AUC by dividing the area into portions of equal width where the area of the trapezium formed is estimated. The sum of these estimations results in the final AUC value.

This study was designed as a complete crossover design with 2 dietary treatments and 2 replicate periods. The Shapiro-Wilk test and the UNIVARIATE procedure in SAS (Version 9.3, SAS Inst. Inc., Cary, NC, USA) were used to verify normality and homogeneity of variances. To compare differences between diets, data were subjected to ANOVA using the MIXED procedure in SAS. Data from the MTT and serum metabolome were analyzed as repeated measures over time with the fixed effects of diet, time, and diet × time. The random effect of replicate period was considered in the main model and individual pig was considered the experimental unit. Differences between least square means were separated using the PDIFF option of SAS and were considered significant if *p* < 0.05 and were described as tendencies if 0.05 ≤ *p* < 0.10. Pearson’s correlation analysis using the PROC CORR procedure of SAS was used to establish and quantify the relationships among blood parameters. Correlations were considered significant if *p* < 0.05.

## 3. Results

### 3.1. Animals and Catheter Function

All animals remained healthy for the duration of the experiment. On blood sampling days, two catheters did not function properly in the first experimental period, and one catheter did not function properly after the first collection time point in the second experimental period. Therefore, fasting data were available for six pigs fed the CON diet and eight pigs fed the TGS diet, whereas a total of six pigs fed the CON diet and seven pigs fed the TGS diet provided postprandial data for analysis.

### 3.2. Serum Metabolomics

Of the 188 metabolites targeted, 133 metabolites were found above the limits of detection with the Absolute*IDQ* p180 kit. This included 21 amino acids, 14 biogenic amines, 5 acylcarnitines, 12 lysophosphatidylcholines, 13 sphingomyelins, 34 diacyl phosphatidylcholines, 33 acyl-alkyl phosphatidylcholines, and the sum of hexoses (Table 2, Table 3 and Table 4 and Table S2). Of the detected metabolites, 10 amino acids, 7 biogenic amines, 4 acylcarnitines, 8 lysophosphatidylcholines, 8 sphingomyelins, 20 diacyl phosphatidylcholines, 14 acyl-alkyl phosphatidylcholines, and the sum of hexoses were affected by TGS consumption at either the overall diet level or at one or more specific time points.

There was a general diet effect on several amino acids that showed higher (*p* < 0.05) concentrations of histidine, lysine, and tryptophan and reduced (*p* < 0.05) concentrations of alanine, aspartate, glutamine, and glutamate as well as a trend (*p* < 0.10) for reduced tyrosine concentrations in serum of pigs fed the TGS diet compared to pigs fed the CON diet (Table 2). Most amino acids followed a similar pattern, in that serum concentrations rose immediately postprandially, and were subsequently reduced by 420 min after meal consumption, thereby approximating or even becoming lower than in the fasting state. In the fasting state, TGS-fed pigs showed reduced (*p* < 0.05) aspartate and glutamate, higher (*p* < 0.05) histidine, and a trend (*p* < 0.10) for higher tryptophan serum concentrations compared to pigs receiving the CON diet. The immediate postprandial phase showed reduced alanine, aspartate, glutamine, and glutamate as well as trends for reduced leucine and valine serum concentrations with the TGS diet compared to the CON diet. Pigs fed the TGS diet showed higher (*p* < 0.05) histidine, lysine, and tryptophan and lower (*p* < 0.05) alanine and glutamate 420 min postprandially compared to pigs fed the CON diet.

Overall diet effects on biogenic amines included elevated (*p* < 0.05) alpha-aminoadipic acid and methionine-sulfoxide, reduced (*p* < 0.05) carnosine and putrescine, and trends for reduced (*p* < 0.10) trans-4-hydroxyproline and taurine concentrations in serum of pigs fed the TGS diet compared to pigs fed the CON diet (Table 2). Nearly all biogenic amines showed a time effect, some increasing and others decreasing postprandially from fasting state levels (Table 2 and Table S2). The effect of TGS consumption on biogenic amines was greater postprandially than in the fasting state. In the fasting state, only alpha-aminoadipic acid was increased by the TGS diet, whereas 30 min postprandially alpha-aminoadipic acid was elevated and putrescine, trans-4-hydroxyproline, and taurine were reduced with TGS consumption compared to the CON diet. Additionally, alpha-aminoadipic acid, methionine-sulfoxide, and sarcosine were all elevated in serum 420 min postprandially with TGS consumption compared to CON.

Sum of hexoses was 17% lower (*p* < 0.05) 30 min postprandially in pigs fed the TGS diet compared to pigs fed the CON diet (Table 3). In the fasting state, serum acylcarnitines showed elevated propionylcarnitine and malonylcarnitine (which remained elevated 30 and 420 min postprandially) as well as trends for elevated free carnitine and myristoleylcarnitine in the fasting state and a trend for elevated free carnitine 420 min postprandially with TGS consumption compared to CON (Table 3 and Table S2).

The greatest effect of TGS consumption was on serum phospholipids (lysophosphatidylcholines, sphingomyelins, and phosphatidylcholines), with over 50% affected by TGS consumption (Table 3 and Table 4 and Table S2). In the fasting state, two lysophosphatidylcholines were increased (lysoC17:0, *p* < 0.05; lysoC20:3, *p* < 0.10) with TGS consumption compared to the CON (Table 3). In contrast, five lysophosphatidylcholines were decreased (lysoC16:1, lysoC18:2, lysoC20:4, *p* < 0.05; lysoC18:0, lysoC18:1, *p* < 0.10) 30 min postprandially and one was decreased 420 min postprandially (lysoC18:2) with consumption of TGS compared to consumption of CON. Of the sphingomyelins detected, serum SM(OH)C14:1, SM(OH)C16:1, SM(OH)C22:1, SM(OH)C24:1, SMC18:0, SMC18:1, and SMC24:1 were all elevated in the fasting state of pigs that consumed the TGS diet compared to pigs that consumed CON (Table 3). No differences between dietary treatments were detected 30 min postprandially, and only one sphingomyelin (SM(OH)C16:1) was elevated 420 min postprandially in TGS-fed pigs compared to those fed the CON diet. The general diet effect on diacyl phosphatidylcholines showed that TGS consumption reduced serum concentrations overall, but predominantly in the postprandial state compared to the CON diet (Table 4). Whereas the overall effects on acyl-alkyl phosphatidylcholines showed that TGS consumption increased certain serum concentrations overall, but mainly in the fasting state compared to consumption of CON (Table 4).

### 3.3. Biochemical Variables

**Figure 1:**
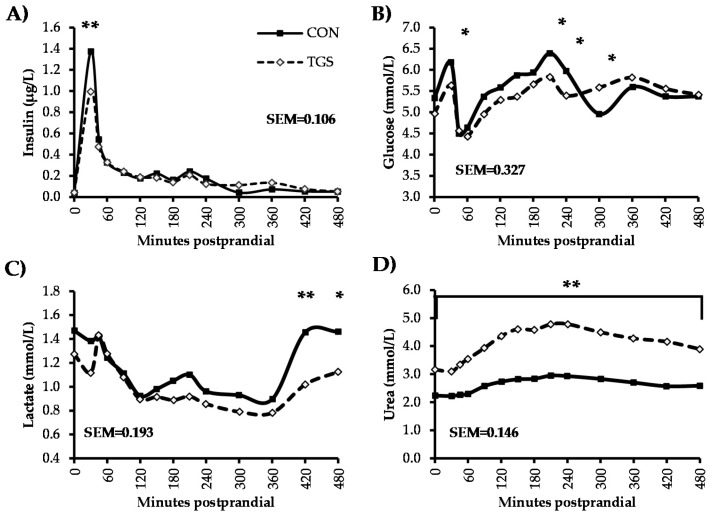
Insulin (**A**); glucose (**B**); lactate (**C**); and urea (**D**) in blood serum or plasma from pigs fed control (CON) or transglycosylated starch (TGS) diet. Data are presented as least square means; control diet *n* = 6, TGS diet *n* = 7. ** CON and TGS diet differ in blood serum or plasma concentrations, *p* < 0.05; * CON and TGS diet tend to differ in blood serum or plasma concentrations, 0.05 < *p* ≤ 0.10.

**Figure 2:**
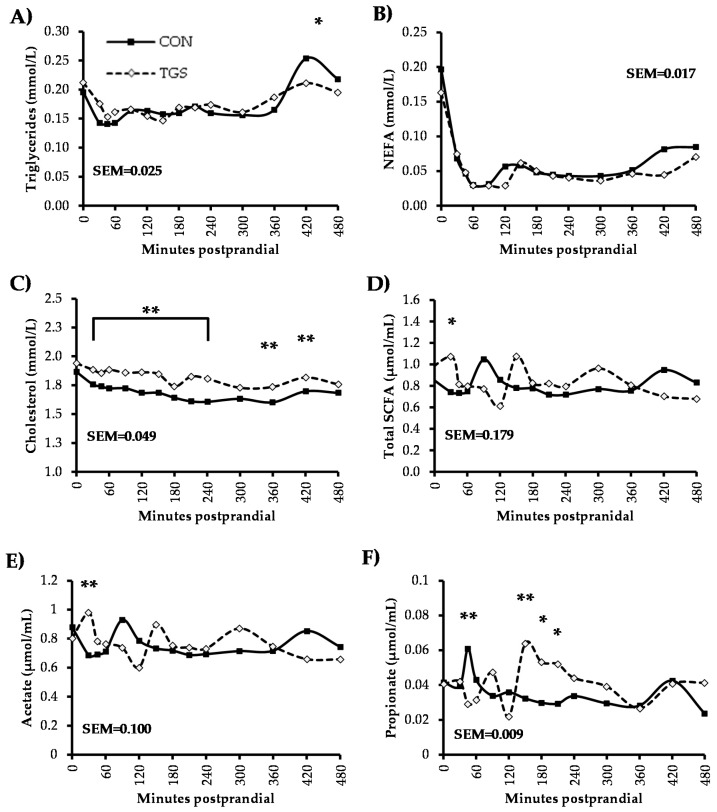
Triglycerides (**A**); non-esterified fatty acids (NEFA; **B**); cholesterol (**C**); total short-chain fatty acids (SCFA; **D**); acetate (**E**); and propionate (**F**) concentrations in blood serum from pigs fed control (CON) or transglycosylated starch (TGS) diet. Data are presented as least square means; control diet *n* = 6, TGS diet *n* = 7. ** CON and TGS diet differ in blood serum or plasma concentrations, *p* < 0.05; * CON and TGS diet tend to differ in blood serum or plasma concentrations, 0.05 < *p* ≤ 0.10.

Fasting concentrations of blood insulin, glucose, lactate, total SCFA, acetate, propionate, triglycerides, and cholesterol were not different between pigs fed the CON and TGS diet (Figure 1 and Figure 2). However, urea concentrations were increased 41% (*p* < 0.05, Figure 1) and NEFA concentrations were reduced 17% (*p* < 0.05, Figure 2) in pigs fed the TGS diet compared to pigs fed the CON diet in the fasting state.

Serum insulin peaked 30 min postprandially with both dietary treatments (Figure 1), and had a 28% lower peak concentration (*p* < 0.05) in pigs fed the TGS diet compared to pigs fed the CON diet. Insulin concentrations then decreased at a similar rate, regardless of dietary treatment, returning to preprandial concentrations about 480 min postprandially. Plasma glucose concentrations followed a similar pattern over time in both dietary treatments, but were generally less variable in pigs fed the TGS diet compared to those fed the CON diet. Plasma glucose concentrations peaked both at 30 and 210 min postprandially, and tended to be approximately 9% lower (*p <* 0.10) at both time points in TGS-fed pigs compared to CON-fed pigs. Plasma glucose concentrations also tended to be 10% lower 240 min postprandially and 13% higher 300 min postprandially in TGS-fed pigs compared to CON-fed pigs before they returned to fasting state levels by 480 min postprandially. Plasma lactate concentrations generally decreased over time in the postprandial phase, regardless of dietary treatment. From 360 to 420 min after feeding, plasma lactate concentrations increased to preprandial levels in pigs fed the CON diet. Although concentrations also were higher in pigs fed the TGS diet 420 min postprandially, they did not reach preprandial levels. Therefore, TGS-fed pigs had about 30% lower serum lactate concentrations 420 min postprandially and tended to have about 23% lower concentrations 480 min postprandially compared to CON-fed pigs. Serum urea concentrations slowly increased from 30 min to 210 min postprandially, then slowly decreased from 240 to 480 min, but did not reach fasting concentrations by the final blood collection time point. Although serum urea in pigs fed both dietary treatments followed a similar pattern over time, concentrations in pigs fed the TGS diet were 40%–60% higher (*p* < 0.05) than those fed the CON diet at every sampling time point. Regardless of dietary treatment, serum triglycerides decreased immediately after feeding (*p* < 0.05) until 420 min postprandially (Figure 2), when concentrations returned to preprandial levels in pigs fed the TGS diet. Serum triglycerides also increased at 420 min for CON-fed pigs, but surpassed preprandial concentrations and tended to be approximately 17% higher than TGS-fed pigs (*p* < 0.10) before returning to preprandial concentrations 480 min postprandially.

Serum NEFA concentrations rapidly decreased (*p* < 0.05) in the immediate postprandial phase until 60 min postprandially regardless of dietary treatment. NEFA concentrations remained low, but slowly began to rise from 360 min postprandially in pigs fed the CON diet, at which point concentrations were 83% higher compared to pigs fed TGS. Serum NEFA did not return to fasting state concentrations during the 8 h MTT. Serum cholesterol concentrations decreased (*p* < 0.05) 30 min postprandially in CON-fed pigs, and remained depressed through the remaining collection time points. Therefore, pigs fed the TGS diet had approximately 6%–13% higher cholesterol concentrations (*p* < 0.05) compared to CON-fed pigs, until the final blood collection time point where no differences were observed between dietary treatments. With both dietary treatments, total serum SCFA concentrations remained relatively constant, showing small peaks over time. The only discernible difference between dietary treatments was 30 min postprandially, where total serum SCFA concentrations increased from the fasting state in TGS-fed pigs, but decreased in CON-fed pigs, which led to increased (*p* < 0.05) total serum SCFA concentrations in TGS-fed pigs compared to CON-fed pigs. With both dietary treatments, serum acetate concentrations showed small peaks over time. Thirty minutes postprandially, serum acetate concentrations were 43%-higher (*p* < 0.05) in TGS-fed pigs compared to CON-fed pigs. Serum propionate concentrations were 52% lower 45 min postprandially in pigs fed the TGS diet compared to pigs fed the CON diet. However, serum propionate increased to nearly 98% greater (*p* < 0.05) concentrations 150 min postprandially in TGS-fed pigs and tended to remain approximately 80% above (*p* < 0.10) CON-fed pigs both 180 and 210 min postprandially before slowly decreasing to fasting state concentrations.

There were no significant effects of dietary treatment (*p* > 0.10) on AUC values (0–480 min) for insulin, glucose, triglycerides, cholesterol, total SCFA, acetate, and propionate (Table 5). However, the AUC value was significantly lower (*p* < 0.05) for lactate, tended to be lower for NEFA (*p* < 0.10), and was significantly higher (*p* < 0.05) for urea in pigs fed the TGS diet compared to those fed CON.

Correlations existed between plasma lactate and serum urea as well as between serum cholesterol and NEFA levels (*p* < 0.001, Table 6). No other correlations existed between the biochemical variables.

### 3.4. Apparent Nutrient Digestibility

Apparent total tract digestibilities of gross energy and dry matter were about 8% lower (*p* < 0.05) in pigs that consumed the TGS diet compared to pigs fed the CON diet (Table 7). Apparent total tract digestibility of protein was approximately 5% lower (*p* < 0.05) in TGS-fed pigs compared to CON-fed pigs. No differences between dietary treatments were observed in ash digestibility at the total tract level.

## 4. Discussion

Despite their wide use in processed foods, data regarding the physiological effects of modified starches are limited. Present results demonstrated the capability of TGS to reduce blood glucose and insulin in the immediate postprandial phase, and to modify protein and lipid metabolism in the fasting state as well as 30 and 420 min postprandially compared to the CON diet. These findings are in general agreement with previously published results from the investigation of resistant and slowly digestible modified starches on glucose and insulin responses [8,9,24] as well as fat metabolism [7,8,11] in humans, pigs, and rodents. It is important to note that actual metabolite fluxes were not measured and that plasma and serum metabolites were determined in a peripheral vein in this study. Therefore, only assumptions can be made about whether metabolite concentrations reflected changes in their uptake, their release, or a combination of the two in response to TGS consumption.

Although AUC values were not different between dietary treatments, the reduced insulin and glucose peaks in the immediate postprandial phase with dietary TGS inclusion indicated a decreased intestinal glucose release. This was supported by the reduced energy and dry matter digestibility, which also suggested hindered intestinal starch digestion in pigs fed the TGS diet. As a result of the acid-catalyzed transglycosylation, endogenous α-amylase should have been unable to cleave many of the rearranged glycosidic bonds, thereby rendering TGS less digestible than the native waxy cornstarch. Considering that plasma glucose and serum insulin were determined in a peripheral vein, postprandial serum insulin concentrations may better correspond to the intestinal release, whereas the postprandial glucose curve may be more reflective of insulin-mediated peripheral glucose regulation. In addition to their direct effect on insulin metabolism, glucose, as well as SCFA, can stimulate the release of satiety-related gut hormones such as peptide YY (PYY; [25,26]), glucagon-like-peptide-1 (GLP-1; [27,28,29]) and glucose-dependent insulinotropic polypeptide (GIP; [29,30]). Although gut incretins were not directly measured in the present study, it is plausible that the greater intestinal generation of propionate with the TGS diet may have stimulated peripheral glucose clearance via gut incretin and insulin secretion (e.g., [31]). Moreover, the reduced intestinal glucose release likely led to a stimulation of glucagon secretion from pancreatic α-cells, which was previously shown for resistant starch [25] but not measured in the present study. A smaller insulin:glucagon ratio may have therefore contributed to an enhanced gluconeogenesis and utilization of propionate in the early postprandial time. The prolonged elevation of propionate in the TGS-fed pigs may have further provided certain satiating effects by stimulating PYY secretion [32,33]. On the other hand, as substrate for gluconeogenesis, elevated serum propionate may have also promoted gluconeogenesis in TGS-fed pigs, thereby increasing plasma glucose levels.

During periods of high glucose availability in the gut, conversion of glucose into L-lactate in the portal drained viscera is proportional to intestinal glucose absorption [15]. Accordingly, in the present study, plasma lactate patterns mirrored those of serum insulin from 60 to 360 min postprandially, before diverging in the present study. Near the end of the MTT (420–480 min), plasma lactate along with serum triglyceride and NEFA concentrations rose, however, less dramatically in TGS-fed pigs than in CON-fed pigs. Purified waxy cornstarch is rapidly and highly digestible [34], therefore, the rise in plasma lactate near the end of the MTT was likely not from fermentation in the hindgut. Instead, it may be the result of its generation in peripheral cells from glycerol molecules left after triglyceride hydrolysis [35]. As glucose availability in the progressing postprandial period becomes limited, other energy sources become increasingly utilized. In our study, serum urea, triglyceride, and NEFA concentrations were elevated throughout or towards the end of the MTT in all pigs, suggesting increased deamination of amino acids via the urea cycle and lipolysis for energy supply [36,37]. Notably, serum urea levels were approximately 30%–40% greater over the entire 8-h blood sampling period in TGS-fed pigs compared to CON, which clearly suggests enhanced utilization of amino acids to balance plasma glucose levels via gluconeogenesis. Casein, the dietary protein source used in our experimental diets, is a high quality, highly digestible source of protein for pigs [14]. As serum metabolite concentrations were determined in a peripheral vein, part of the absorbed amino acids were obviously directly used for hepatic gluconeogenesis in pigs fed the TGS diet in the early postprandial phase, which was supported by the serum amino acid concentrations 30 min postprandially. Elevated serum lysine in combination with reduced glucogenic amino acids (i.e., alanine, aspartate, glutamate, glutamine, and tyrosine) in the TGS-fed pigs 30 min postprandially supports that amino acids were being directed towards maintaining energy balance in the body in place of protein biosynthesis. Lysine is the main amino acid involved in protein biosynthesis and has been shown to be the most conserved [38,39], whereas alanine and glutamate are key amino acids balancing glucose and nitrogen in the body [40]. Increased serum urea and lower amino acid concentrations in the fasting state and late postprandial period (420 min) may have been related to an enhanced amino acid release from muscle protein in TGS-fed pigs compared to CON-fed pigs. However, urea was inversely correlated with lactate in the present study, indicating that amino acid deamination was reduced when alternative energy sources were available. Additionally, alpha-aminoadipate, which was the only biogenic amine that was affected consistently at all three time points, was significantly greater in TGS-fed pigs compared to CON-fed pigs. Since alpha-aminoadipate is formed via the degradation of lysine [41], it is conceivable that in spite of the higher serum lysine concentration with the TGS diet, lysine was increasingly degraded in pigs fed the TGS diet when compared to pigs fed the CON diet. Increased blood tryptophan levels, which were seen in TGS-fed pigs 420 min postprandially and in the fasting state when compared to CON-fed pigs, may suggest an increased release from muscle protein. Tryptophan is a precursor to serotonin, which has been shown to alter behavior and overall digesta transit time [42,43]. If digesta transit time was modified with TGS consumption, it may have affected the progression of digestion and therefore energy supply throughout the MTT period.

It is generally assumed that cholesterol levels are reduced when less digestible starch sources are included in the diet due to increased fecal bile acid secretion in combination with changes in mRNA levels of key enzymes in cholesterol metabolism pathways [44]. Contrary to findings with dietary inclusion of an enzymatically modified starch product [7], cholesterol levels were increased postprandially in the present study with TGS consumption. In this study, we did not discriminate between the various fractions of cholesterol; therefore it is not possible to know which fractions were raised or lowered in our pigs, and thus whether the observed cholesterol levels would implicate positive or negative effects on cardiovascular health in humans [45].

In order to guarantee a continuous energy supply to the body, carnitine transports long-chain fatty acids from the cytosol to the mitchondria for fatty acid oxidation, and acylcarnitines are synthesized via carnitine palmitoyltransferase 1 (CPT1; [46]). Incomplete, overloaded, or upregulated lipid oxidation can lead to accumulations of acylcarnitines [47,48]. In our study, the higher levels of free carnitine, propionylcarnitine, and malonylcarnitine in combination with lower serum triglyceride and NEFA concentrations in the fasting state and 420 min postprandially with TGS consumption suggest that lipid oxidation rates in TGS-fed pigs may have been incomplete or overloaded compared to CON-fed pigs. A previous study investigating the effects of an enzymatically modified starch on serum metabolome of pigs in the pre- and postprandial states found that long-chain lysophosphatidylcholines, glycerophospholipids, and sphingolipids were not influenced by meal consumption, but were modified by dietary EMS inclusion similarly in the fasting state and 60 min postprandially [7]. Our study showed numerous differences in lysophosphatidylcholines, glycerophospholipids, and sphingolipids between the fasting and two postprandial states (30 and 420 min), indicating that meal consumption played a role in their fluxes. With the exception of a single sphingomyelin (C26:0), every glycerophospholipid, lysophospholipid, and sphingomyelin decreased 30 min postprandially in TGS-fed pigs and increased 30 min postprandially in CON-fed pigs compared to fasting state levels, although many did not reach significance. This would suggest that there is a direct link between TGS consumption and serum concentrations of these phospholipids. These phospholipid groups are critical structural components of plasma lipoproteins and cell membranes and have roles in membrane protein trafficking, regulation of cell function, and inflammation [49]. Certain phospholipids have been linked to obesity and insulin resistance [50,51,52]. The greatest number of differences in phosphatidylcholines and lysophosphatidylcholines between dietary treatments occurred in the immediate postprandial phase (30 min). Considering the effects that insulin has on lipid metabolism via modulating the expression of transcription factors related to fatty acid synthesis in the liver [53], it is likely that insulin played a significant role. High insulin concentrations were previously suggested to lead to decreased plasma concentrations of sphingolipids, lysophosphatidylcholines, and phospholipids [51]. However, present results suggested the opposite. It is also possible that gut hormones such as GLP-1 and PYY, which were not directly measured in the present study, and the changes in the absorbed SCFA (e.g., acetate) could have played a role in the large changes in postprandial phospholipid profiles [43,54].

Since the pig is regarded as a reliable model to study digestive physiology and metabolic responses [12,13], it can be reasonably assumed that the present trial can provide some insight into the potential effects of dietary TGS inclusion on human health. Still, translation of the results must be done with care since human diets will contain much less TGS than fed to the pigs in the present study. Particularly, the observed effect of TGS to reduce blood glucose and insulin in the immediate postprandial phase as well as its effect on phospholipid metabolism may be favorable for moderating insulin resistance [50,51,52].

## 5. Conclusions

In conclusion, our results indicated a potential for TGS to beneficially alter postprandial insulin and glucose metabolism, thereby causing alterations in serum amino acid and phospholipid metabolome. Specifically, TGS consumption altered the metabolism of sphingolipids and phospholipids, in both the fasting and postprandial state. These effects may aid in the prevention or moderation of certain health conditions, such as insulin resistance and obesity, in humans. However, further research will need to be conducted to confirm the effects of TGS at lower rates of dietary inclusion in more complex human diets.

## Figures and Tables

**Table 1:** Ingredient and analyzed nutrient composition of experimental diets.

Item	CON Diet	TGS Diet ^1^
Ingredient composition, %		
Waxy cornstarch	72.10	36.05
Transglycosylated cornstarch	0.00	36.05
Casein	18.00	18.00
Lignocellulose ^2^	4.00	4.00
Rapeseed oil	1.00	1.00
Monocalcium phosphate	4.00	4.00
Vitamin-mineral premix ^3^	0.60	0.60
Titanium dioxide	0.30	0.30
Analyzed nutrient composition (dry matter basis, g/kg)		
Gross energy (MJ/kg)	17.8	17.8
Dry matter	944	956
Crude protein	179	174
Total starch	728	709
Calcium	7.4	7.6
Phosphorus	4.5	4.6

Analyzed nutrient composition presented on a dry matter basis. ^1^ CON, control starch; TGS, transglycosylated cornstarch (ARIC, Tulln, Austria); ^2^ FibreCell (agromed Austria GmbH, Austria); ^3^ Provided per kilogram of complete diet (GARANT GmbH, Austria): 16,000 IU of vitamin A, 2000 IU of vitamin D_3_, 125 mg of vitamin E, 2.0 mg of vitamin B_1_, 6.0 mg of vitamin B_2_, 3.0 mg of vitamin B_6_, 0.03 mg of vitamin B_12_, 3.0 mg of vitamin K_3_, 30 mg of niacin, 15.0 mg of pantothenic acid, 900 mg of choline chloride, 0.15 mg of biotin, 1.5 mg of folic acid, 200 mg of vitamin C; 4.6 g of Ca, 2.3 g as digestible P, 2.4 g as Na, 2.0 g of Cl, 3.2 g K, 1.0 g Mg; 50 mg of Mn (as MnO); 100 mg of Zn (as ZnSO_4_); 120 mg of Fe (as FeSO_4_), 15.6 mg of Cu (as CuSO_4_), 0.5 mg of Se (as Na_2_SeO_3_), 1.9 mg of I (as Ca(IO_3_)_2_).

**Table 2:** Selected serum amino acids and biogenic amines (μmol/L) of pigs fed transglycosylated (TGS) or control (CON) starch diets pre- and postprandially.

Metabolite	Fasting State		30 min Postprandially		420 min Postprandially		Pooled SEM	*p*-Value		
	CON	TGS	CON	TGS	CON	TGS		Diet	Time	D × T
Amino acids										
Alanine	589.5 ^cd^	540.1 ^d^	838.6 ^a^	721.4 ^b^	645.0 ^bcA^	539.2 ^cdB^	45.23	0.006	<0.001	0.586
Arginine	160.1 ^bc^	166.1 ^b^	229.5 ^a^	212.0 ^a^	147.1 ^bc^	127.1 ^c^	12.53	0.306	<0.001	0.482
Asparagine	81.6 ^c^	79.1 ^c^	146.5 ^a^	138.1 ^ab^	123.9 ^b^	125.2 ^b^	7.32	0.532	<0.001	0.709
Aspartate	23.9 ^a^	15.0 ^b^	24.1 ^a^	12.7 ^b^	17.4 ^ab^	11.5 ^b^	3.16	0.001	0.199	0.627
Citrulline	84	90.8	94.2	86.6	103.6	98.7	11.75	0.778	0.188	0.609
Glutamine	962 ^cd^	884 ^d^	1139 ^a^	1008 ^bc^	1086 ^ab^	990 ^bd^	58.9	0.008	0.002	0.797
Glutamate	138.1 ^b^	106.6 ^c^	168.0 ^a^	118.3 ^bc^	133.8 ^b^	97.2 ^c^	11.77	<0.001	0.008	0.539
Glycine	1710	1805 ^a^	1825 ^ad^	1822 ^ab^	1519 ^c^	1537 ^cd^	128.4	0.671	0.014	0.864
Histidine	42.3 ^d^	69.4 ^c^	104.8 ^a^	116.6 ^a^	86.0 ^b^	115.0 ^a^	7.09	<0.001	<0.001	0.233
Isoleucine	177.2 ^c^	178.8 ^c^	303.5 ^a^	282.4 ^a^	228.5 ^b^	225.4 ^b^	13.66	0.497	<0.001	0.65
Leucine	238.2 ^c^	248.1 ^c^	446.6 ^aA^	401.0 ^aB^	333.2 ^b^	322.4 ^b^	18.59	0.314	<0.001	0.308
Lysine	322.1 ^d^	366.7 ^d^	599.8 ^a^	628.4 ^a^	435.3 ^c^	518.8 ^b^	31.22	0.016	<0.001	0.493
Methionine	44.0 ^b^	47.8 ^b^	105.7 ^a^	97.7 ^a^	92.9 ^a^	96.3 ^a^	5.12	0.946	<0.001	0.336
Ornithine	121.9 ^c^	128.0 ^c^	182.6 ^a^	168.1 ^ab^	145.6 ^bc^	155.6 ^b^	10.63	0.946	<0.001	0.347
Phenylalanine	55.9 ^c^	62.3 ^c^	142.5 ^a^	131.6 ^a^	112.1 ^b^	116.4 ^b^	6.38	0.995	<0.001	0.203
Proline	361.9 ^b^	373.1 ^b^	655.9 ^a^	602.4 ^a^	607.1 ^a^	649.4 ^a^	29.83	0.999	<0.001	0.11
Serine	161.5 ^c^	158.4 ^c^	252.3 ^a^	237.8 ^ab^	215.7 ^b^	220.8 ^b^	10.63	0.568	<0.001	0.503
Threonine	209.6 ^c^	247.3 ^bc^	311.9 ^a^	305.0 ^a^	281.7 ^ab^	302.2 ^a^	21.99	0.277	<0.001	0.445
Tryptophan	41.8 ^dB^	50.8 ^dA^	87.5 ^a^	85.7 ^ab^	63.6 ^c^	77.0 ^b^	4.22	0.023	<0.001	0.073
Tyrosine	221.4 ^c^	196.3 ^c^	330.2 ^ab^	290.0 ^b^	349.7 ^a^	325.3 ^ab^	32.93	0.059	<0.001	0.87
Valine	487.6 ^c^	478.4 ^c^	672.6 ^aA^	611.1 ^abB^	586.1^b^	566.7 ^b^	25.22	0.105	<0.001	0.412
Biogenic amines										
α-AAA	15.7 ^c^	32.1 ^a^	12.2 ^cd^	26.5 ^b^	8.2 ^d^	14.4 ^c^	2.63	<0.001	<0.001	0.006
Carnosine	16.2	14.5	16.2	13.9	17	14.4	1.82	0.043	0.848	0.939
Met-SO	3.6 ^d^	3.4 ^d^	7.1 ^c^	6.5 ^c^	9.5 ^b^	12.8 ^a^	0.52	0.037	<0.001	0.001
Putrescine	0.72 ^a^	0.66 ^ac^	0.70 ^a^	0.57 ^bcd^	0.60 ^abd^	0.54 ^d^	0.043	0.027	0.03	0.611
Sarcosine	1.1 ^b^	1.2 ^b^	1.5 ^b^	0.9 ^b^	1.7 ^b^	3.1 ^a^	0.4	0.335	0.002	0.041
t4-OH-Pro	95.1 ^bc^	91.8 ^bc^	112.7 ^a^	96.6 ^b^	87.0 ^bc^	81.1 ^c^	6.12	0.053	0.001	0.379
Taurine	30.9 ^bc^	32.1 ^b^	44.8 ^a^	27.2 ^bcd^	20.3 ^c^	17.3 ^d^	4.05	0.052	<0.001	0.045

SEM, standard error of the mean; D × T, Diet × Time; α-AAA, α-amino adipate; Met-SO, methionine-sulfoxide; t4-OH-Pro, trans-4-hydroxyproline. All values are presented as least square means ± SEM; CON diet, *n* = 6; TGS diet, *n* = 7. Means with different superscript letters within a row differed significantly (*p* < 0.05) over all sampling time points. Means with different capital superscript letters tended to differ (0.05 < *p* ≤ 0.10) over all sampling time points.

**Table 3:** Sum of hexoses and selected acylcarnitines, lysophosphatidylcholines and sphingomyelins (μmol/L) of pigs fed transglycosylated (TGS) or control (CON) starch diets pre- and postprandially.

Metabolite	Fasting State		30 min Postprandial		420 min Postprandial		Pooled SEM	*p*-Value		
	CON	TGS	CON	TGS	CON	TGS		Diet	Time	D × T
Sum of hexoses	6204 ^b^	6155 ^b^	7526 ^a^	6283 ^b^	6496	6249 ^b^	550.8	0.115	0.170	0.272
Acylcarnitines										
C0	3.0 ^BD^	3.2 ^aAC^	3.2 ^AB^	3.0 ^bCD^	3.1	3.2	0.13	0.677	0.704	0.040
C3	0.04 ^b^	0.06 ^a^	0.05 ^b^	0.07 ^a^	0.04 ^b^	0.07 ^a^	0.004	<0.001	0.292	0.874
C3-DC	0.05 ^b^	0.09 ^a^	0.05 ^b^	0.10 ^a^	0.05 ^b^	0.11 ^a^	0.010	<0.001	0.438	0.645
Lysophosphatidylcholines										
lysoPC a C16:1	1.7 ^ab^	1.8 ^ab^	1.8 ^a^	1.5 ^bc^	1.5 ^bc^	1.3 ^c^	0.15	0.110	0.005	0.117
lysoPC a C17:0	0.46 ^b^	0.61 ^a^	0.51 ^b^	0.50 ^b^	0.45 ^b^	0.49 ^b^	0.069	0.047	0.178	0.073
lysoPC a C18:1	21.0	22.8 ^a^	23.2 ^aA^	19.8 ^B^	19.7	17.6 ^b^	1.71	0.287	0.032	0.130
lysoPC a C18:2	6.3 ^ab^	5.4 ^bc^	6.9 ^a^	4.9 ^c^	6.2 ^ab^	4.5 ^c^	0.63	<0.001	0.336	0.316
lysoPC a C20:3	1.7 ^B^	2.0 ^aA^	1.8	1.7	1.5 ^b^	1.5 ^b^	0.12	0.473	0.028	0.237
lysoPC a C20:4	4.1 ^ab^	3.8 ^ab^	4.3 ^a^	3.3 ^bc^	3.6	2.9^c^	0.56	0.011	0.058	0.541
Sphingomyelins										
SM (OH) C14:1	1.2 ^b^	1.4 ^a^	1.3	1.2 ^b^	1.2 ^b^	1.3	0.10	0.109	0.721	0.108
SM (OH) C16:1	1.5 ^c^	1.9 ^a^	1.6 ^bc^	1.7	1.5 ^c^	1.8 ^ab^	0.21	<0.001	0.651	0.247
SM (OH) C24:1	0.19 ^b^	0.25 ^a^	0.22	0.21	0.21	0.21	0.024	0.334	0.942	0.187
SM C18:0	7.5 ^b^	9.0 ^a^	7.8 ^b^	8.0	7.6 ^b^	8.2	0.61	0.037	0.626	0.282
SM C18:1	2.0 ^b^	2.5 ^a^	2.1 ^b^	2.2	2.0 ^b^	2.3	0.19	0.026	0.544	0.384
SM C24:1	9.2 ^b^	10.5 ^a^	9.8	9.6	9.7	9.8	0.55	0.308	0.951	0.198
SM C26:0	0.03 ^bc^	0.04 ^b^	0.06 ^ab^	0.07 ^a^	0.01 ^c^	0.03 ^b^	0.011	0.089	0.002	0.810

SEM, standard error of the mean; D × T, Diet × Time; C0, free carnitine; C3, propionylcarnitine; C3-DC, malonylcarnitine; lysoPC a, lysophosphatidylcholine with acyl residue C; SM (OH) C, hydroxysphingomyelin with acyl residue sum C; SM C, sphingomyelin with acyl residue sum C. Values are presented as least square means ± SEM; control diet, *n* = 6; TGS diet, *n* = 7. Means with different superscript letters within a row differed significantly (*p* < 0.05) over all sampling time points. Means with different capital superscript letters tended to differ (0.05 < *p* ≤ 0.10) over all sampling time points.

**Table 4:** Selected phosphatidylcholines (μmol/L) of pigs fed transglycosylated (TGS) or control (CON) starch diets pre- and postprandially.

Metabolite	Fasting State		30 min Postprandial		420 min Postprandial		Pooled SEM	*p*-Value		
	CON	TGS	CON	TGS	CON	TGS		Diet	Time	D × T
PC aa C32:0	7.2 ^ab^	6.6 ^bc^	7.6 ^a^	5.9 ^c^	7.9 ^a^	6.0 ^c^	0.49	<0.001	0.812	0.063
PC aa C32:2	0.23 ^ab^	0.20 ^abc^	0.24 ^a^	0.15 ^cd^	0.16 ^bcdA^	0.09 ^dB^	0.040	0.005	0.003	0.393
PC aa C32:3	0.05 ^b^	0.06	0.07 ^a^	0.06 ^b^	0.07	0.06 ^b^	0.006	0.305	0.357	0.065
PC aa C34:1	203 ^bc^	231 ^a^	214 ^ab^	208 ^ab^	179 ^c^	174 ^c^	16.2	0.510	<0.001	0.140
PC aa C34:2	70.8 ^ab^	65.3 ^bc^	76.1 ^a^	61.0 ^c^	77.3 ^a^	58.7 ^c^	5.44	<0.001	0.983	0.127
PC aa C34:3	3.1 ^abc^	2.9 ^bcd^	3.4 ^a^	2.7 ^cd^	3.3 ^ab^	2.5 ^d^	0.30	<0.001	0.478	0.192
PC aa C36:2	80.0 ^ab^	76.6 ^ab^	85.5 ^a^	70.1 ^bc^	73.9 ^b^	61.8 ^c^	4.79	0.003	0.010	0.227
PC aa C36:6	0.13 ^ab^	0.12 ^ab^	0.14 ^aA^	0.11 ^B^	0.10 ^bc^	0.09 ^c^	0.017	0.089	0.008	0.611
PC aa C38:0	0.65	0.64	0.72 ^a^	0.62	0.67	0.57 ^b^	0.076	0.080	0.533	0.518
PC aa C38:3	34.8 ^bcd^	42.6 ^a^	37.1 ^bc^	39.1 ^ab^	30.6 ^d^	33.3 ^cd^	3.25	0.019	0.002	0.259
PC aa C38:4	84.3 ^ab^	85.1 ^ab^	88.5 ^aA^	76.6 ^B^	72.0 ^bc^	65.2 ^c^	9.43	0.148	0.003	0.387
PC aa C40:2	0.50 ^ab^	0.44 ^bc^	0.55 ^a^	0.40 ^c^	0.49 ^ab^	0.39 ^c^	0.074	<0.001	0.391	0.189
PC aa C40:3	1.1 ^b^	1.1 ^b^	1.3 ^a^	1.1 ^b^	1.1 ^b^	0.9 ^c^	0.16	0.009	0.010	0.153
PC aa C40:4	4.6 ^ab^	4.1 ^abc^	4.7 ^a^	3.6 ^cd^	3.9 ^bc^	3.1 ^d^	0.33	0.001	0.004	0.372
PC aa C40:5	13.4 ^ab^	12.5 ^ab^	14.0 ^a^	11.2 ^bc^	11.3 ^bc^	9.6 ^c^	1.11	0.008	0.003	0.404
PC aa C42:1	0.05 ^bcCD^	0.06 ^abAB^	0.06 ^aAC^	0.05 ^BD^	0.05 ^bc^	0.04 ^c^	0.004	0.438	0.038	0.035
PC aa C42:2	0.11 ^abA^	0.10 ^bcB^	0.12 ^a^	0.09 ^c^	0.11 ^a^	0.10 ^c^	0.005	<0.001	0.934	0.346
PC aa C42:4	0.09	0.10	0.11 ^a^	0.08 ^b^	0.10	0.08 ^b^	0.007	0.070	0.747	0.126
PC aa C42:5	0.16 ^ab^	0.16 ^ab^	0.18 ^a^	0.14 ^bc^	0.17 ^a^	0.13 ^c^	0.010	<0.001	0.408	0.016
PC aa C42:6	0.27 ^ab^	0.25 ^ab^	0.29 ^a^	0.23 ^bc^	0.25 ^ab^	0.20 ^c^	0.016	0.003	0.027	0.298
PC ae C32:1	1.4	1.5	1.6 ^a^	1.3 ^b^	1.5	1.3 ^b^	0.09	0.044	0.812	0.102
PC ae C34:0	0.63	0.68 ^a^	0.66	0.61	0.67 ^a^	0.58 ^b^	0.058	0.303	0.551	0.048
PC ae C34:1	6.6 ^B^	7.5 ^aA^	6.9	6.6 ^b^	6.5 ^b^	6.2 ^b^	0.42	0.692	0.096	0.109
PC ae C36:0	0.61 ^b^	0.79 ^a^	0.72 ^a^	0.79 ^a^	0.76 ^a^	0.77 ^a^	0.047	0.005	0.127	0.037
PC ae C36:1	6.3 ^bc^	8.2 ^a^	6.6 ^b^	7.6 ^a^	5.5 ^cB^	6.4 ^bcA^	0.63	<0.001	<0.001	0.206
PC ae C38:1	0.9 ^c^	1.1 ^a^	1.0 ^ab^	1.1 ^ab^	0.9 ^bc^	1.0 ^ab^	0.07	0.004	0.291	0.051
PC ae C38:2	1.4 ^c^	1.7 ^a^	1.5 ^bcB^	1.6 ^abA^	1.3 ^c^	1.5 ^bc^	0.10	<0.001	0.021	0.140
PC ae C38:3	2.4 ^c^	3.1 ^a^	2.5 ^bcBD^	2.9 ^abAC^	2.2 ^cCD^	2.5 ^bcAB^	0.21	<0.001	0.006	0.238
PC ae C38:5	4.6	4.6	5.0 ^A^	4.3 ^B^	5.0	4.3	0.44	0.085	0.987	0.323
PC ae C40:3	0.61 ^b^	0.69	0.67	0.68	0.59 ^b^	0.71 ^a^	0.069	0.022	0.697	0.327
PC ae C42:2	0.25 ^bcd^	0.33 ^a^	0.27 ^abc^	0.30 ^ab^	0.20 ^d^	0.23 ^cd^	0.033	0.014	0.001	0.279
PC ae C42:3	0.18 ^B^	0.22 ^aA^	0.21 ^ab^	0.22 ^a^	0.17 ^c^	0.17 ^b^	0.023	0.247	0.006	0.286
PC ae C42:4	0.13	0.15	0.15 ^A^	0.12 ^bB^	0.13	0.16 ^a^	0.012	0.832	0.769	0.036
PC ae C44:3	0.05 ^b^	0.06	0.07 ^aA^	0.05 ^B^	0.06	0.06	0.009	0.626	0.626	0.068

SEM, standard error of the mean; D × T, Diet × Time; PC aa C, phosphatidylcholine with diacyl residue sum C; PC ae C, phosphatidylcholine with acyl-alkyl residue sum C. Values are presented as least square means ± SEM; control diet, *n* = 6; TGS diet, *n* = 7. Means with different superscript letters within a row differed significantly (*p* < 0.05) over all sampling time points. Means with different capital superscript letters tended to differ (0.05 < *p* ≤ 0.10) over all sampling time points.

**Table 5:** Area under the curve values (0–480 min) during serial blood samplings of pigs fed transglycosylated (TGS) or control (CON) starch diets.

Metabolite	CON	TGS	SEM	*p*-Value
Insulin (mol/L × 480 min)	100	89	11.7	0.386
Glucose (μg/L × 480 min)	2669	2582	70.9	0.250
Lactate (mmol/L × 480 min)	544	452	40.3	0.045
Urea (mmol/L × 480 min)	1230	2057	234.8	0.006
Triglycerides (mmol/L × 480 min)	83	83	6.9	0.946
NEFA (mmol/L × 480 min)	28	23	2.4	0.067
Cholesterol (mmol/L × 480 min)	843	840	49.6	0.958
SCFA (μmol/mL × 480 min)	393	395	32.3	0.963
Acetate (μmol/mL × 480 min)	365	363	44.8	0.962
Propionate (μmol/mL × 480 min)	17	19	7.1	0.347

SEM, standard error of the mean; NEFA, non-esterified fatty acids; SCFA, short-chain fatty acids. Values are presented as least square means ± SEM; control diet, *n* = 6; TGS diet, *n* = 7.

**Table 6:** Relationship between blood parameters of pigs fed control and transglycosylated starch diets.

Parameters	Correlation Coefficient, r	*p*-Value
Lactate: Urea	−0.426	<0.001
Cholesterol: NEFA	0.308	<0.001

NEFA, non-esterified fatty acids: All other correlations were not significant.

**Table 7:** Apparent total tract digestibility of pigs fed transglycosylated (TGS) or control (CON) starch diets.

Item, %	CON	TGS	SEM	*p*-Value
Dry matter	93.0	85.5	0.56	<0.001
Gross energy	93.2	85.4	0.53	<0.001
Crude protein	95.4	90.2	0.64	<0.001
Crude ash	70.5	72.2	0.87	0.188

Values are presented as least square means ± SEM; *n* = 8 per dietary treatment.

## Supplementary Materials

The following are available online at http://www.mdpi.com/2072-6643/9/3/291/s1, Table S1: Concentrations of standards used for SCFA analysis and Table S2: Additional serum metabolites (μmol/L) of pigs fed transglycosylated (TGS) or control (CON) starch diets pre- and postprandially.

## Abbreviations

The following abbreviations are used in this manuscript:

AUC	Area under the curve
CON	Control starch
NEFA	Non-esterified fatty acids
SCFA	Short-chain fatty acids
TGS	Transglycosylated starch
