# Supplementary material for: Transglycosylated Starch Improves Insulin Response and Alters Lipid and Amino Acid Metabolome in a Growing Pig Model
Source: Nutrients. 2017 Mar 16;9(3):291. doi: 10.3390/nu9030291 (PMC5372954; doi:10.3390/nu9030291)
Supplement: Supplementary file 1 [file nutrients-09-00291-s001.docx]

**Newman et al. - Supplementary Materials**

| **Table S1. Concentration of SCFA standards used for gas chromatography analysis^1^** | | |
| --- | --- | --- |
| **SCFA** | **Concentration (µmol/mL)** | **Total in vial (µmol)** |
| Acetic acid | 2.62 | 1.05 |
| Propionic acid | 1.34 | 0.54 |
| Iso-butyric acid | 0.27 | 0.11 |
| Butyric acid | 0.54 | 0.22 |
| Iso-valeric acid | 0.23 | 0.09 |
| Valeric acid | 0.23 | 0.09 |
| Internal standard (4-methyl-valeric acid) | 0.79 | 0.08 |
| Caproic acid | 0.20 | 0.08 |

^1^Calculations according to the equation: density = mass / volume.

**Table S2.** Additional serum metabolites (µmol/L) of pigs fed transglycosylated (TGS) or control (CON) starch diets pre- and postprandially.

| **Metabolite, µmol/L** | **Fasting state** | | | | |  | | **30 min postprandial** | |  | **420 min postprandial** | | **Pooled SEM** | ***P*-value** | | | | | |  |
| --- | --- | --- | --- | --- | --- | --- | --- | --- | --- | --- | --- | --- | --- | --- | --- | --- | --- | --- | --- | --- |
|  | **CON** | | | **TGS** | |  | | **CON** | **TGS** |  | **CON** | **TGS** |  | **Diet** | **Time** | | | | **D × T** |  |
| **Biogenic amines** |  | | | |  |  | |  |  |  |  |  |  |  |  | | | |  |  |
| ADMA | | | 2.0 | | 1.7^b^ |  | | 2.2 | 2.3^a^ |  | 1.8 | 1.7 | 0.29 | 0.628 | 0.052 | | | | 0.662 |  |
| Creatinine | | | 84.7 | | 89.5 |  | | 92.5 | 87.5 |  | 95.8 | 94.2 | 5.90 | 0.899 | 0.356 | | | | 0.666 |  |
| Histamine | | | 0.41 | | 0.62^a^ |  | | 0.31^b^ | 0.41 |  | 0.21^b^ | 0.17^b^ | 0.145 | 0.279 | 0.004 | | | | 0.382 |  |
| Kynurenine | | | 0.15^c^ | | 0.52 |  | | 0.42^bc^ | 0.30^bc^ |  | 1.12^a^ | 0.86^ab^ | 0.218 | 0.974 | 0.009 | | | | 0.290 |  |
| Serotonin | | | 3.4^a^ | | 2.6^ab^ |  | | 2.0^bc^ | 1.5^bc^ |  | 1.1^c^ | 1.4^bc^ | 0.55 | 0.408 | 0.002 | | | | 0.387 |  |
| Spermidine | | | 0.18 | | 0.19^ab^ |  | | 0.22^a^ | 0.19^ab^ |  | 0.17^bc^ | 0.14^c^ | 0.017 | 0.182 | 0.017 | | | | 0.503 |  |
| SDMA | | | 0.72^bc^ | | 0.69^c^ |  | | 0.83^ab^ | 0.86^a^ |  | 0.67^c^ | 0.63^c^ | 0.044 | 0.749 | <0.001 | | | | 0.731 |  |
| **Acylcarnitines** | | |  | |  |  | |  |  |  |  |  |  |  |  | | | |  |  |
| C2 | | | 0.30 | | 0.32 |  | | 0.35 | 0.34 |  | 0.34 | 0.31 | 0.018 | 0.554 | 0.175 | | | | 0.400 |  |
| C14:1 | | | 0.03^B^ | | 0.03^A^ |  | | 0.03 | 0.03 |  | 0.03 | 0.03 | 0.002 | 0.481 | 0.469 | | | | 0.237 |  |
| **Lysophosphatidylcholines** | | | | | |  | |  |  |  |  |  |  |  |  | | | |  |  |
| lysoPC a C16:0 | | | 21.1 | | 23.7^a^ |  | | 22.4 | 20.3 |  | 21.2 | 19.5^b^ | 1.93 | 0.705 | 0.285 | | | | 0.141 |  |
| lysoPC a C18:0 | | | 10.9 | | 11.5 |  | | 12.4^aA^ | 10.6^B^ |  | 10.7 | 9.9^b^ | 1.01 | 0.220 | 0.161 | | | | 0.178 |  |
| lysoPC a C26:0 | | | 0.04 | | 0.06 |  | | 0.09 | 0.05 |  | 0.08 | 0.06 | 0.019 | 0.570 | | 0.320 | | | 0.287 |  |
| lysoPC a C26:1 | | | 0.03 | | | 0.04 |  | 0.06 | | 0.03 |  | 0.05 | 0.05 | 0.012 | 0.477 | | 0.344 | | 0.382 | |
| lysoPC a C28:0 | | | 0.08 | | | 0.11 |  | 0.12^A^ | | 0.08^B^ |  | 0.11 | 0.11 | 0.018 | 0.820 | | 0.633 | | 0.068 | |
| lysoPC a C28:1 | | | 0.05 | | | 0.09 |  | 0.13 | | 0.08 |  | 0.12 | 0.10 | 0.030 | 0.602 | | 0.314 | | 0.373 | |
| **Sphingomyelins** | | |  | |  |  | |  |  |  |  |  |  |  |  | |  | | |  |
| SM (OH) C22:1 | | | 1.8 | | 1.9 |  | | 1.9 | 1.7 |  | 1.8 | 1.7 | 0.25 | 0.741 | 0.289 | | 0.357 | | |  |
| SM (OH) C22:2 | | | 1.1^b^ | | 1.3^a^ |  | | 1.2 | 1.1^b^ |  | 1.1^b^ | 1.2 | 0.10 | 0.209 | 0.706 | | 0.095 | | |  |
| SM C16:0 | | | 41.8 | | 45.6 |  | | 43.2 | 40.4 |  | 42.7 | 41.5 | 3.19 | 0.984 | 0.613 | | 0.262 | | |  |
| SM C16:1 | | | 4.2 | | 4.6 |  | | 4.4 | 4.1 |  | 4.2 | 4.2 | 0.35 | 0.835 | 0.712 | | 0.305 | | |  |
| SM C20:2 | | | 0.12 | | 0.13 |  | | 0.14 | 0.12 |  | 0.12 | 0.13 | 0.012 | 0.939 | 0.851 | | 0.689 | | |  |
| SM C24:0 | | | 7.4 | | 7.9 |  | | 7.6 | 7.1 |  | 7.0 | 6.9 | 0.47 | 0.990 | 0.170 | | 0.396 | | |  |
| **Phosphatidylcholines** | | |  | |  |  | |  |  |  |  |  |  |  |  | |  | | |  |
| PC aa C24:0 | | | 0.10 | | 0.15 |  | | 0.21 | 0.13 |  | 0.15 | 0.18 | 0.052 | 0.956 | 0.674 | | 0.390 | | |  |
| PC aa C28:1 | | | 0.40 | | 0.45 |  | | 0.47 | 0.41 |  | 0.44 | 0.44 | 0.040 | 0.917 | 0.904 | | 0.194 | | |  |
| PC aa C30:0 | | | 1.4 | | 1.5^a^ |  | | 1.5 | 1.3^b^ |  | 1.4 | 1.3^b^ | 0.11 | 0.304 | 0.336 | | 0.133 | | |  |
| PC aa C32:1 | | | 8.6^ab^ | | 9.5^a^ |  | | 8.8^ab^ | 8.1^bc^ |  | 6.7^cd^ | 6.1^d^ | 0.87 | 0.807 | <0.001 | | 0.216 | | |  |
| PC aa C34:4 | | | 0.17^a^ | | 0.18^a^ |  | | 0.18^a^ | 0.17^a^ |  | 0.15 | 0.13^b^ | 0.022 | 0.485 | 0.008 | | 0.289 | | |  |
| PC aa C36:0 | | | 1.3^ab^ | | 1.3^ab^ |  | | 1.3^a^ | 1.2 |  | 1.1^bc^ | 1.1^c^ | 0.08 | 0.243 | 0.018 | | 0.573 | | |  |
| PC aa C36:1 | | | 70.5^a^ | | 75.9^a^ |  | | 73.7^a^ | 67.9^a^ |  | 58.5^b^ | 55.9^b^ | 4.39 | 0.726 | <0.001 | | 0.188 | | |  |
| PC aa C36:3 | | | 33.4 | | 36.6^a^ |  | | 35.7 | 33.1 |  | 34.6 | 31.5^b^ | 2.86 | 0.594 | 0.491 | | 0.140 | | |  |
| PC aa C36:4 | 42.4 | | | | 46.9^a^ |  | | 44.9^ab^ | 41.6 |  | 38.0^bc^ | 36.0^c^ | 5.47 | 0.896 | 0.008 | | 0.242 | | |  |
| PC aa C36:5 | 3.0^ab^ | | | | 3.2^a^ |  | | 3.3^a^ | 2.9 |  | 2.5^bc^ | 2.4^c^ | 0.40 | 0.539 | 0.002 | | 0.358 | | |  |
| PC aa C38:1 | 0.05^b^ | | | | 0.05^b^ |  | | 0.11 | 0.10 |  | 0.14^a^ | 0.08 | 0.030 | 0.285 | 0.072 | | 0.456 | | |  |
| PC aa C38:5 | 28.1^ab^ | | | | 29.1^ab^ |  | | 30.1^a^ | 26.1 |  | 24.6^bc^ | 22.7^c^ | 2.78 | 0.293 | 0.014 | | 0.338 | | |  |
| PC aa C38:6 | 11.3 | | | | 12.5^a^ |  | | 12.1^ab^ | 11.2 |  | 9.9^bc^ | 9.3^c^ | 1.72 | 0.903 | 0.009 | | 0.324 | | |  |
| PC aa C40:6 | 14.0^ab^ | | | | 14.6^a^ |  | | 14.7^a^ | 13.2^ab^ |  | 11.8^bc^ | 10.7^c^ | 1.71 | 0.333 | <0.001 | | 0.329 | | |  |
| PC ae C30:0 | 0.37 | | | | 0.40^a^ |  | | 0.39 | 0.35^b^ |  | 0.39 | 0.38 | 0.021 | 0.517 | 0.621 | | 0.179 | | |  |
| PC ae C32:2 | 0.30 | | | | 0.33 |  | | 0.35 | 0.31 |  | 0.32 | 0.31 | 0.017 | 0.521 | 0.580 | | 0.148 | | |  |
| PC ae C34:2 | 3.0 | | | | 3.3 |  | | 3.3 | 3.1 |  | 3.4 | 3.1 | 0.26 | 0.579 | 0.871 | | 0.182 | | |  |
| PC ae C34:3 | 1.1 | | | | 1.2 |  | | 1.2 | 1.1 |  | 1.3 | 1.2 | 0.13 | 0.367 | 0.553 | | 0.182 | | |  |
| PC ae C36:2 | 4.8 | | | | 5.3^a^ |  | | 5.2 | 4.8 |  | 4.7 | 4.6^b^ | 0.31 | 0.870 | 0.238 | | 0.202 | | |  |
| PC ae C36:3 | 2.0 | | | | 2.2 |  | | 2.1 | 2.0 |  | 2.1 | 2.0 | 0.15 | 0.566 | 0.999 | | 0.160 | | |  |
| PC ae C36:4 | 3.0 | | | | 3.1 |  | | 3.2 | 2.8 |  | 3.1 | 2.8 | 0.31 | 0.189 | 0.820 | | 0.356 | | |  |
| PC ae C36:5 | 1.5 | | | | 1.6 |  | | 1.6 | 1.5 |  | 1.5 | 1.4 | 0.12 | 0.976 | 0.500 | | 0.303 | | |  |
| PC ae C38:0 | 0.58 | | | | 0.58^a^ |  | | 0.62^a^ | 0.56 |  | 0.49 | 0.46^b^ | 0.056 | 0.408 | 0.017 | | 0.702 | | |  |
| PC ae C38:4 | 5.3 | | | | 5.8^a^ |  | | 5.6 | 5.2 |  | 5.1 | 4.9^b^ | 0.54 | 0.876 | 0.171 | | 0.271 | | |  |
| PC ae C38:6 | 1.0 | | | | 1.0 |  | | 1.1 | 0.9 |  | 1.0 | 0.9 | 0.11 | 0.204 | 0.772 | | 0.186 | | |  |
| PC ae C40:1 | 0.50 | | | | 0.56^a^ |  | | 0.50 | 0.55^a^ |  | 0.41^b^ | 0.43^b^ | 0.076 | 0.130 | 0.011 | | 0.610 | | |  |
| PC ae C40:2 | 0.39 | | | | 0.40 |  | | 0.43 | 0.38 |  | 0.39 | 0.40 | 0.025 | 0.669 | 0.854 | | 0.456 | | |  |
| PC ae C40:4 | 1.5 | | | | 1.5 |  | | 1.6^a^ | 1.4 |  | 1.4 | 1.4^b^ | 0.08 | 0.459 | 0.212 | | 0.382 | | |  |
| PC ae C40:5 | 1.2 | | | | 1.3^a^ |  | | 1.3^a^ | 1.1 |  | 1.2 | 1.1^b^ | 0.10 | 0.301 | 0.150 | | 0.399 | | |  |
| PC ae C40:6 | 1.1 | | | | 1.2 |  | | 1.2 | 1.1 |  | 1.1 | 1.0 | 0.14 | 0.556 | 0.391 | | 0.226 | | |  |
| PC ae C42:1 | 0.30 | | | | 0.35^a^ |  | | 0.35^a^ | 0.33 |  | 0.28 | 0.26^b^ | 0.036 | 0.893 | 0.024 | | 0.315 | | |  |
| PC ae C44:5 | 0.17 | | | | 0.18 |  | | 0.18 | 0.17 |  | 0.17 | 0.17 | 0.013 | 0.860 | 0.858 | | 0.261 | | |  |
| PC ae C44:6 | 0.10 | | | | 0.11 |  | | 0.11 | 0.10 |  | 0.10 | 0.10 | 0.021 | 0.754 | 0.542 | | 0.471 | | |  |

SEM, standard error of the mean; D × T, Diet × Time; ADMA, asymmetric dimethylarginine; SDMA, symmetric dimethylarginine; C2, acetylcarnitine; C14:1, tetradecenoylcarnitine; lysoPC a, lysophosphatidylcholine with acyl residue C; SM (OH) C, hydroxysphingomyelin with acyl residue sum C; SM C, sphingomyelin with acyl residue sum C; PC aa C, phosphatidylcholine with diacyl residue sum C; PC ae C, phosphatidylcholine with acyl-alkyl residue sum C. Values are presented as least square means ± SEM; control diet, n = 6; TGS diet, n = 7. Means with different superscript letters within a row differed significantly (*P*<0.05) over all sampling time points. Means with different capital superscript letters tended to differ (0.05<*P*≤0.10) over all sampling time points.
